# Liver fibrosis staging with a new 2D-shear wave elastography using comb-push technique: Applicability, reproducibility, and diagnostic performance

**DOI:** 10.1371/journal.pone.0177264

**Published:** 2017-05-16

**Authors:** Sang Min Lee, Jeong Min Lee, Hyo-Jin Kang, Hyung Kung Yang, Jeong Hee Yoon, Won Chang, Su Joa An, Kyoung Bun Lee, Seung Yon Baek

**Affiliations:** 1 Department of Radiology, Seoul National University Hospital, Seoul, Korea; 2 Institute of Radiation Medicine, Seoul National University College of Medicine, Seoul, Korea; 3 Department of Pathology, Seoul National University Hospital, Seoul, Korea; 4 Department of Radiology, School of Medicine, Ewha Womans University, Seoul, Korea; Yonsei University College of Medicine, REPUBLIC OF KOREA

## Abstract

**Objective:**

To evaluate the applicability, reproducibility, and diagnostic performance of a new 2D-shear wave elastography (SWE) using the comb-push technique (2D CP-SWE) for detection of hepatic fibrosis, using histopathology as the reference standard.

**Materials and methods:**

This prospective study was approved by the institutional review board, and informed consent was obtained from all patients. The liver stiffness (LS) measurements were obtained from 140 patients, using the new 2D-SWE, which uses comb-push excitation to produce shear waves and a time-aligned sequential tracking method to detect shear wave signals. The applicability rate of 2D CP-SWE was estimated, and factors associated with its applicability were identified. Intraobserver reproducibility was evaluated in the 105 patients with histopathologic diagnosis, and interobserver reproducibility was assessed in 20 patients. Diagnostic performance of the 2D CP-SWE for hepatic fibrosis was evaluated by receiver operating characteristic (ROC) curve analysis.

**Results:**

The applicability rate of 2D CP-SWE was 90.8% (109 of 120). There was a significant difference in age, presence or absence of ascites, and the distance from the transducer to the Glisson capsule between the patients with applicable LS measurements and patients with unreliable measurement or technical failure. The intraclass correlation of interobserver agreement was 0.87, and the value for the intraobserver agreement was 0.95. The area under the ROC curve of LS values for stage F2 fibrosis or greater, stage F3 or greater, and stage F4 fibrosis was 0.874 (95% confidence interval [CI]: 0.794–0.930), 0.905 (95% CI: 0.832–0.954), and 0.894 (95% CI: 0.819–0.946), respectively.

**Conclusion:**

2D CP-SWE can be employed as a reliable method for assessing hepatic fibrosis with a reasonably good diagnostic performance, and its applicability might be influenced by age, ascites, and the distance between the transducer and Glisson capsule.

## Introduction

Chronic liver disease (CLD) is a serious health concern worldwide, and infections associated with hepatitis B virus (HBV) and hepatitis C virus (HCV), alcohol abuse, and non-alcoholic fatty liver disease (NAFLD) are the common predisposing conditions for developing liver fibrosis and cirrhosis [1]. Without proper management of CLD, it progresses to liver fibrosis and consequently leads to liver cirrhosis, which increases morbidity and mortality owing to portal hypertension, hepatic insufficiency, and hepatocellular carcinoma (HCC) [2, 3]. As the prognosis and treatment of CLD vary depending on the stage of fibrosis, the most important question for the clinician is whether the patient with CLD has cirrhosis [4, 5]. However, the diagnosis of compensated liver cirrhosis is quite challenging [1]. Moreover, several studies have demonstrated that antiviral therapy in viral hepatitis enables regression of fibrosis [6–8], and the preference for antiviral therapy in patients with chronic HBV and HCV infections is driven by the presence or absence of moderate to severe fibrosis [1]. Currently, liver biopsy is considered as the gold standard method for stratification of hepatic fibrosis [9, 10]. However, liver biopsy is an invasive procedure and has limitations of sampling error and variability of histologic interpretation [1,11, 12]. Further, it is not feasible in a routine clinical setting to monitor liver fibrosis with repeated liver biopsy [1, 13].

Therefore, various noninvasive tests for assessment of liver fibrosis have emerged, which include serum biomarkers (e.g., AST to Platelet Ratio Index [14]) and various elastographic techniques [15–18]. Among the elastographic techniques, transient elastography (TE), a vibroacoustic non-imaging technology, has been most extensively evaluated, and its good reproducibility and good diagnostic performance for detection of significant hepatic fibrosis have been demonstrated [19, 20]. However, it has limitations such as a small region-of-interest (ROI) that cannot be selected, no B-mode orientation, and limited applicability for some patients with increased body mass index (BMI) and ascites [19, 21]. Although magnetic resonance elastography (MRE) is widely accepted for providing excellent diagnostic accuracy for fibrosis staging with the largest sampling volume [1, 18], it is expensive and less readily available than ultrasound-based shear wave elastography (US-SWE) [18]. Recently, several major ultrasound manufacturers implemented either point SWE (pSWE) technique measuring the speed of shear wave in a small region (a few millimeters) or two-dimensional (2D)-SWE techniques measuring shear wave velocity in a bidimensional area (in a range of 2~3cm per side), in their clinical US systems [22, 23]. These SWE techniques have an advantage of placing ROI under real-time imaging and have been reported to have a comparable diagnostic performance for diagnosing significant fibrosis to TE [1, 18, 24]. The first commercially available 2D-SWE technique (Aixplorer; SuperSonic Imagine S.A., Aix-en-Provence, France), SSI 2D-SWE uses shear wave generated from multiple focused push beams at different depths in tissue and equips with software beamformer enabling high pulse-repetition-frequency (PRF) to track shear wave. Therefore, SSI 2D-SWE can provide advantages of a larger ROI than TE or pSWE, and a possibility of real-time measurement with a color display of liver stiffness (LS) values [1, 17, 18, 25–28]. However, 2D-SWE could not be implemented on conventional diagnostic ultrasound system because of its low PRF to track shear waves. More recently, a new 2D-SWE using comb-push ultrasound shear elastography (2D CP-SWE) to produce and process multiple shear waves and time-aligned sequential tracking to allow conventional ultrasound system with low PRF to track shear wave signals by sequentially exciting vectors in a imaging zone has been developed and implemented on a commercial ultrasound machine (LOGIQ E9; General Electric [GE]) [29, 30]. This new 2D CP-SWE has the merit of rapid and solid recontruction of a large full FOV elasticity maps with the only single acquisition by generating multiple shear wave from multiple unfocused push beams with a comb pattern followed by directional filtering to remove the interferences, which enables real-time monitoring and minimizing motion artifact [29, 30]. However, there have not been sufficient data to validate its diagnostic performance for staging for hepatic fibrosis using liver biopsy as the standard of reference. Therefore, the purpose of this study was to evaluate the applicability, reproducibility, and diagnostic performance of the comb-push 2D-SWE in the detection of hepatic fibrosis, using histopathology as the reference standard.

## Materials and methods

This prospective study was approved by the Institutional Review Board (IRB) of Seoul National University Hospital, and written informed consent was obtained from all patients (NCT02673411).

### Study population

**Fig 1:**
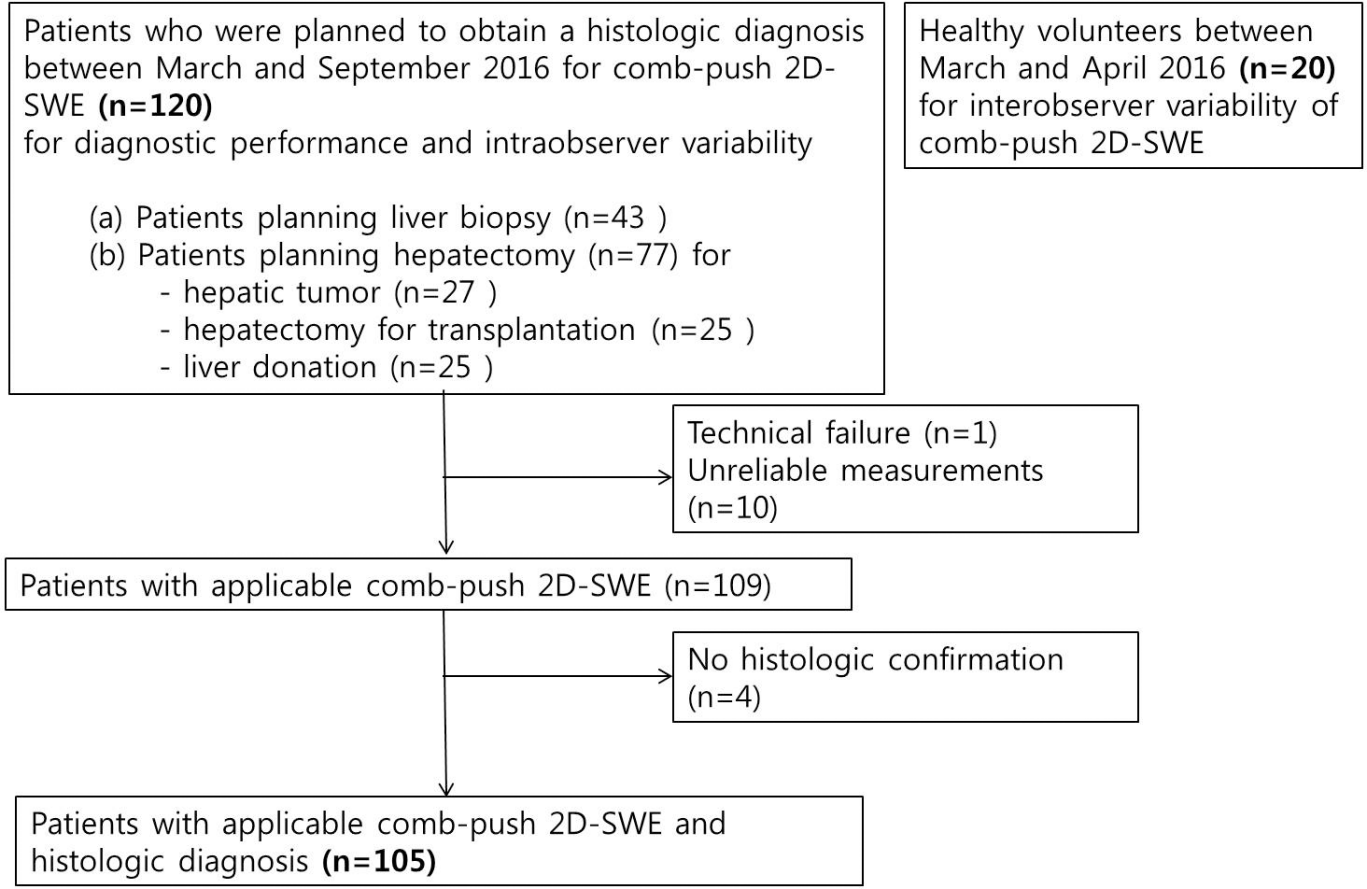
Flow diagram of the study population.

Between March and October 2016, 120 patients (77 men, 43women; mean age, 52.2 years±13.40 [standard deviation]; age range, 19–78 years) who planned to obtain a histologic diagnosis of liver fibrosis were included in this study (Fig 1). The eligibility criteria were as follows: (a) patients referred to the radiology department for liver parenchymal biopsy who were suspected of having a chronic liver disease or scheduled for biopsy after liver transplantation to exclude recurrent hepatitis and (b) patients who planned to undergo hepatectomy for hepatic tumor, transplantation, or liver donation. Patients younger than 18 years were not included in this study. The BMIs of all patients were recorded (mean BMI, 23.7 kg/m^2^ ± 2.98 [standard deviation]; range, 16.5–31.3 kg/m^2^).

In addition, 20 healthy volunteers (7 men, 13 women; mean age, 52.8 years± 8.38[standard deviation]; age range, 36–69 years, BMI <25 kg/m^2^) were included for analysis of interobserver reproducibility. All volunteers were older than 18 years and had no biochemical or ultrasonographic features of liver cirrhosis.

### Measurements of liver stiffness values by using the 2D CP-SWE technique

**Fig 2:**
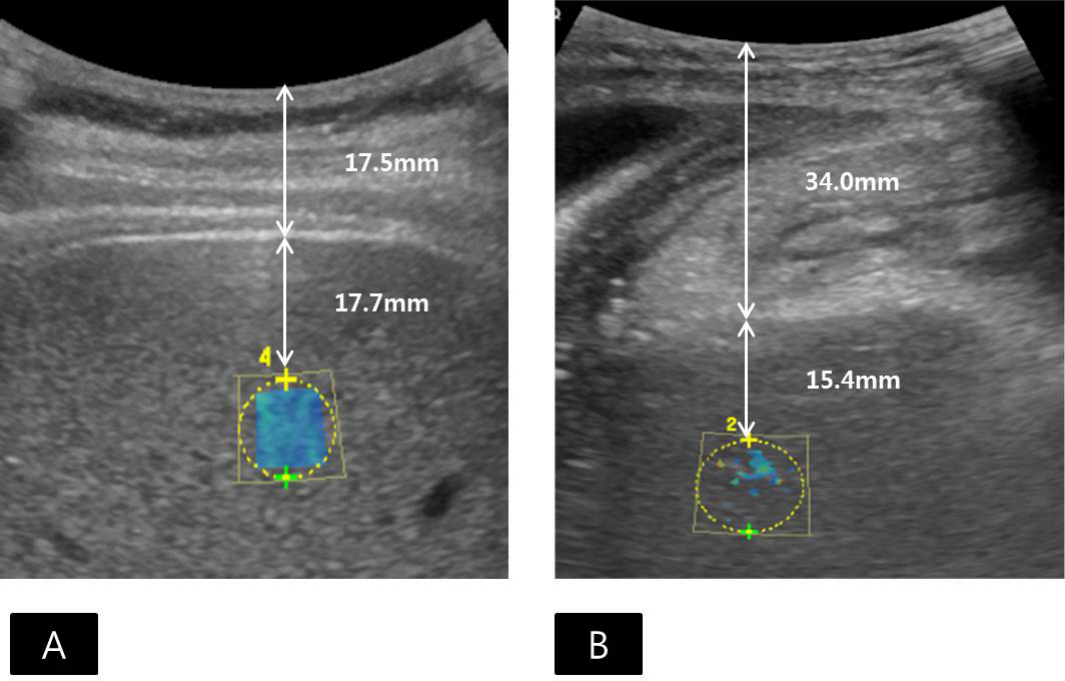
Applicability of 2D comb-push shear wave elastography (SWE) measurements. (A) Applicable SWE examination with technical success and reliable measurement in a 43-year-old male liver donor with a body mass index (BMI) of 25.73 kg/m^2^. The region of interest (ROI) was placed in the right upper lobe, avoiding large vessels and areas with artifacts, and 17.7mm away from the Glisson capsule. Sufficient color maps covering more than 50% of the sampling area for all acquisitions were obtained, which was regarded as a technical success. The interquartile range/median ratio was 6.51%, which was lower than 30% and was considered as a reliable measurement. The median liver stiffness (LS) value was 4.15kPa, and the histologic fibrosis was proven to be stage F0. Note that the distance between the transducer and Glisson capsule was 17.5mm. (B) Non-applicable SWE examination with technical failure in a 68-year-old female patient with a BMI of 21.26kg/m^2^,who was awaiting liver transplantation. The ROI was placed in the right upper lobe, devoid of large vessels and areas of artifacts, and 15.4mm away from the Glisson capsule. Despite 12 trials, color filling of the elastography map was insufficient, which was regarded as a technical failure. Note that this was the only case of technical failure in our study, and the distance between the transducer and Glisson capsule was 34.0mm.

2D CP-SWE was performed using customized GE LOGIQ E9 (GE Healthcare, Wauwatosa, WI, USA) on a high frame mode with a convex probe (C1-6 probe) through the intercostal approach. One of three radiologists (S.M.L, H.J.K, H.K.Y), who had nine years, five years, and five years of experience in abdominal ultrasonography respectively, measured LS values 12 times in the first session and 12 times in the second session to check for intraobserver variability. 2D-SWE measurements in 20 volunteers were successively evaluated by two radiologists (S.M.L and W.C with nine years and six years of experience in abdominal ultrasonography, respectively) for interobserver variability, each of whom measured 12 sequential LS values. The B-mode scan was intervened by two sessions for sequential LS measurements. The interval between two sessions was less than 20 minutes. All patients who had fasted for over 6 hours were placed in the supine position, with their right arm extended above the head. A 1 × 1-cm^2^ region-of-interest (ROI) was placed in the right lobe of the liver, particularly the right upper lobe, avoiding large vessels and areas with artifacts, 1.5–2.0cm away from the Glisson capsule, and less than 6cm deep from the capsule (Fig 2) [1, 31]. During the same session, the ROI was put at the same location in the liver. For obtaining a cine loop of 2D-SWE, patients were asked to hold their breath for each measurement lasting less than 5 seconds. After obtaining the cine loop of the colored elasticity maps with a frame rate of one per second during the breath-holding, the most stable elasticity map with a homogenous color filling in the ROI was selected by an operator from the cine loop. Measurement of LS was automatically done by placing a circular ROI in the center portion of the acquisition ROI.

Technical failure of 2D CP-SWE was defined as failure to obtain a color map in more than 50% of the sampling area for all acquisitions [30]. If the interquartile range (IQR)/median ratio of 12 LS measurement values was higher than 30%, the result was regarded as unreliable measurement [21]. The median LS values and IQR/median ratio of the 12 measurements for each session were calculated. The applicability rate was defined as the ratio of exams that showed technical success and reliable measurement to all exams [32]. In addition, the distance between the transducer and Glisson capsule, and the distance between the Glisson capsule and ROI was recorded.

### Histologic analysis

The specimens obtained through surgery or biopsy were fixed in a formalin–alcohol–acetic acid solution and embedded in paraffin. Subsequently, the specimens were cut into 4-mm-thick slices and staining with hematoxylin–eosin. All specimens were analyzed by an expert liver pathologist (K.B.L, with 10 years of experience in the interpretation of hepatic pathologic examination) who was not blinded to the 2D CP-SWE and clinical data. According to the METAVIR scoring system, liver necroinflammatory activity grade (A grade) and fibrosis stage (F stage) were assessed [10, 19]. Fibrosis was graded from F0 to F4 (F0, no fibrosis; F1, portal fibrosis; F2, periportal fibrosis; F3, septal fibrosis; and F4, cirrhosis). Advanced fibrosis was defined as fibrosis of stage greater than stage F2. Steatosis was classified as absent (S0), less than 5% (S1), 5%–33% (S2), 34%–66% (S3), and more than 66% (S4). Necroinflammatory activity grade consisted of lobular activity and periportal activity, which were graded from 0 to 4: Grade A0 indicated no activity, Grade A1 indicated minimal activity, Grade A2 indicated mild activity, Grade A3 indicated moderate activity, and Grade A4 indicated severe activity.

### Statistical analysis

Statistical analyses were performed using commercially available software programs (SPSS, version 23; SPSS, IBM, Armonk, NY, USA; or MedCalc, version 16, MedCalcSoftware, Mariakerke, Belgium) and a free software program (R version 2.6.0; R Package for Statistical Computing, *www.r-project.org*). To determine the difference between applicable 2D CP-SWE and non-applicable 2D CP-SWE, we used the Mann–Whitney *U* test for continuous variables and Fisher’s exact test for categorical variables. Interobserver and intraobserver agreements were evaluated using intraclass correlation (ICC) and Pearson correlation for 2D CP-SWE results. Spearman correlation test was performed to determine whether the fibrosis, steatosis, and necroinflammation staging classified by METAVIR system were correlated with LS values [33]. For this, multiple regression analysis was performed. To evaluate the differences of possible confounders (age, sex, BMI, etiology of chronic liver disease, the presence of ascites, steatosis, necroinflammation) and fibrosis among patients with high LS values, univariate and multivariate analysis were performed. The continuous variables such as age and BMI were dichotomized around median values. The histologic variables were divided as follows: fibrosis (F0-1 and F2-4), steatosis (S0-2 and S3-4), and necroinflammation (A0-2 and A3-4). For the dichotomized variables, the χ^2^ test was used for univariate analysis and then multivariate analysis using logistic regression model for all parameters showing P≥0.2 in univariate analysis was performed to adjust for potential confounder and to predict the LS values.

To assess the diagnostic performance of 2D CP-SWE in differentiating (a) fibrosis of F1 or greater (stage ≥F1), (b) fibrosis of F2 or greater (stage ≥F2), (c) fibrosis of F3 or greater (stage ≥F3), and (d) fibrosis F4, a nonparametric receiver operating characteristic (ROC) curve was plotted. To minimize spectrum bias, the adjusted area under the curve (AUC) was calculated using the standardization method through the difference between advanced (F2-4) and non-advanced (F0-1) fibrosis stages (DANA) proposed by Poynard et al.[31, 34]. As the gold standard is not binary scale, the overall diagnostic performance of 2D CP-SWE was obtained as the Obuchowski measure, using R package [35].

## Results

### Applicability rate of 2D CP-SWE and factors associated with its applicability

Technical failure of 2D CP-SWE was observed in one patient (1/120, 0.8%), and unreliable measurement of 2D CP-SWE was seen in 10 patients (10/120, 8.3%), yielding 90.8% (109/120) of the applicability rate (Fig 1). There was a significant difference in age, presence or absence of ascites, and the distance from the transducer to the Glisson capsule between the patients with applicable LS measurements and patients with unreliable measurement or technical failure (Table 1). The mean age of patients with non-applicable 2D CP-SWE measurements (mean age, 60.3±12.3years) was significantly higher than that of patients with applicable 2D CP-SWE measurements (mean age, 51.4±13.3 years) (P = 0.028). There were 12 patients with ascites, and six of them had non-applicable LS measurements, while the other six had applicable measurements. In 12 patients with ascites, the age, sex, BMI, the distance between transducer and Glisson capsule, and the distance between Glisson capsule and ROI didn't significantly differ according to the applicability of LS measurements (S1 Table). The histologic fibrosis stages in all patients with ascites were proven to be F4. The distance between the transducer and Glisson capsule in patients with in-applicable 2D CP-SWE measurements (mean, 27.0 ±15.1 mm) was significantly greater than that in patients with applicable 2D CP-SWE measurements (mean, 16.8 ±5.9 mm, P = 0.011, Fig 2). However, there was no significant difference in sex distribution, BMI, or the distance between Glisson capsule and ROI between patients with applicable 2D CP-SWE and non-applicable 2D CP-SWE (Table 1).

**Table 1:** Comparison between patients with applicable 2D CP-SWE and patients with non-applicable 2D CP-SWE.

	Applicable 2D CP-SWE (n = 109)	Non-applicable 2D CP-SWE (n = 11)	P value
Mean age	51.4±13.3	60.3±12.3	0.028
Sex*			0.198
M	72 (66.1%)	5 (45.5%)	
F	37 (33.9%)	6 (54.5%)	
Mean BMI(kg/m^2^)	23.7±3.0	23.9±3.0	0.989
Ascites*	6 (5.5%)	6 (54.5%)	< 0.001
The distance between transducer and Glisson capsule (mm)	16.8±5.9	27.0±15.1	0.011
The distance between Glisson capsule and ROI (mm)	16.6±5.0	13.7±5.0	0.092

2D CP-SWE, 2-dimensional comb-push shear wave elastography; BMI, body mass index; ROI, region-of-interest

Note.-Unless otherwise indicated, data are means ± standard deviations.

*Data are numbers of patients, and data in parentheses are percentages.

### Histologic findings in patients included in the final analysis

Of the 109 patients with applicable 2D CP-SWE, 105 patients who underwent liver biopsy (n = 39) or surgery (n = 66) were included in the final analysis after excluding four patients (2 patients waiting for liver transplantation and two liver donor candidates) who eventually did not undergo surgery (Fig 1). The median interval between 2-D SWE and histologic confirmation was one day (range, 0–60 days). The histologic diagnoses are listed in S2 Table. Among the 105 patients, the etiology of 54 patients with chronic hepatitis is described in S3 Table. The clinical characteristics and METAVIR scorings of 105 patients with applicable 2D CP-SWE and histologic diagnosis are summarized in Table 2. In each fibrosis stage except for F4, LS values did not show correlation with BMI (S4 Table). In fibrosis staging F4, LS showed mild correlation with BMI with intermediate statistical significance (r = -0.484, 95% CI: -0.776–0.022, P = 0.042).

**Table 2:** Clinical characteristics and METAVIR scorings of 105 patients with applicable 2D CP-SWE and histologic diagnosis.

**Mean age**	51.6±13.3 (19–78)
**Sex**	
M	69 (65.7%)
F	36 (34.3%)
**Mean BMI(kg/m**^**2**^**)**	23.7±3.0 (16.5–31.3)
BMI<25 (normal)	71 (67.6%)
25≤BMI<30 (overweight)	32 (30.5%)
BMI≥30 (obese)	2 (1.9%)
**Ascites**	5 (4.8%)
**Fibrosis**	
F0	44 (41.9)
F1	17 (16.2)
F2	12 (11.4)
F3	14 (13.3)
F4	18 (17.1)
**Steatosis**	
S0	51 (48.6)
S1	12 (11.4)
S2	31 (29.5)
S3	10 (9.5)
S4	1 (1.0)
**Necroinflammation**	
A0	26 (24.8)
A1	69 (65.7)
A2	8 (7.6)
A3	2 (1.9)

2D CP-SWE, 2-dimensional comb-push shear wave elastography; BMI, body mass index

Note.- Unless otherwise indicated, data area number and data in parentheses are percentages.

**Fig 3:**
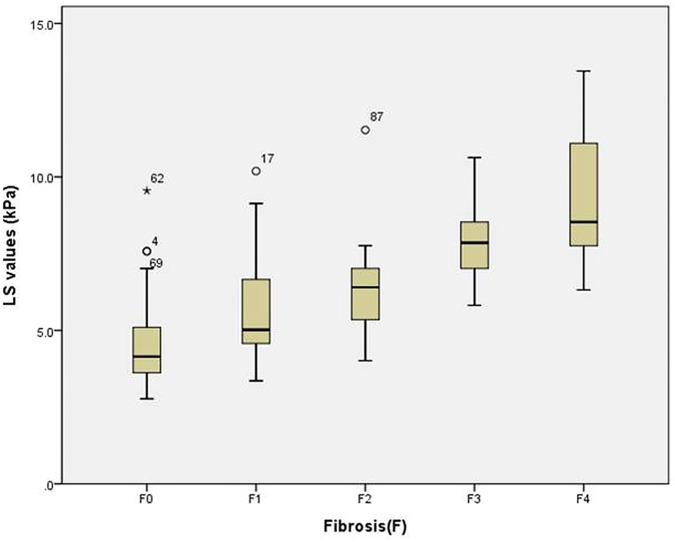
Box-and-whisker plot shows the liver stiffness (LS) values at each fibrosis staging. The boxes represent LS values from the 25^th^ to 75^th^ quartile, and the lines at the center of the boxes indicate the median. The whiskers represent the 9^th^ and 91^st^ percentiles. LS values are expressed in kilopascals.

LS values measured with the 2D CP-SWE showed high correlation with estimation of fibrosis (r = 0.713, 95% CI: 0.603–0.796, P<0.001, Fig 3) and mild correlation with necroinflammation (r = 0.448, 95% CI: 0.281–0.589, P<0.001). However, hepatic steatosis did not show correlation with LS values (r = 0.048, 95% CI: −0.145–0.238, P = 0.625). Subsequent multiple regression analysis for these continuous values revealed that fibrosis staging (β = 1.123, P<0.001) was an independent factor affecting LS values (R^2^ = 0.506, P<0.001).

### Interobserver and intraobserver reliability of 2D CP-SWE

**Fig 4:**
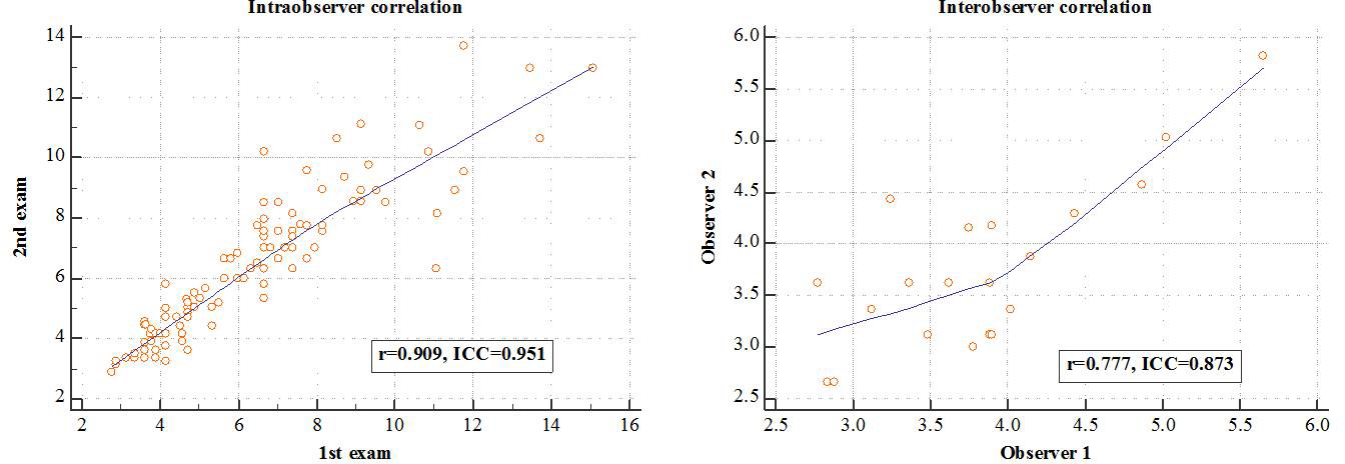
Scatterplots show good intra-and interobserver correlations for 2D comb-push shear wave elastography. The Pearson correlation, r and the intra-class correlation, ICC are shown in the plot.

The ICC for intraobserver agreement of LS measurements using the 2D CP-SWE for 105 patients with applicable 2D CP-SWE and histologic diagnosis was 0.95 (95% CI: 0.93–0.97), which represented an excellent correlation (r = 0.91, Fig 4). Furthermore, the reproducibility of LS measurements with 2D CP-SWE between observers in 20 volunteers yielded an ICC of 0.87 (95% CI: 0.68–0.95), which represented a high correlation (r = 0.78).

When an ICC for each histologic fibrosis stage was obtained to determine whether intraobserver variability tended to increase as fibrosis staging, the ICCs for F0, F1, F2, F3, and F4 were 0.97 (95% CI: 0.94–0.98), 0.83 (95% CI: 0.54–0.94), 0.93 (95% CI: 0.76–0.98), 0.92 (95% CI: 0.75–0.97), and 0.86 (95% CI: 0.62–0.95), respectively. There was no increasing tendency of the intraobserver variability as the fibrosis staging increases.

### Univariate and multivariate analysis for affecting factors and LS values

Factors associated with LS values in univariate and multivariate analyses are listed in Table 3. In univariate analysis, age greater than 54 years (OR4.0, 95% CI 1.78–9.03, P = 0.001), presence of ascites (OR 1.1, 95% CI 1.01–1.19, P = 0.035), fibrosis staging greater than F2 (OR 38.4, 95% CI 10.44–141.56, P<0.001), and chronic hepatitis B (HBV-CLD) (OR 20.6, 95% CI 5.73–74.29) were significantly associated with the high LS values (>5.33kPa). Through multivariate analysis, fibrosis staging greater than F2 (OR 21.7, 95% CI 2.90–162.47, P = 0.003) was an independently associated with the high LS values (>5.33kPa). Age greater than 54 years (OR 5.2, 95% CI 1.65–16.54, P = 0.005) showed statistically intermediate association with the high LS values.

**Table 3:** The predictors for high LS value (>5.33kPa) which is the cutoff value for differentiating advanced fibrosis (≥F2) from non-advanced fibrosis.

	Univariate			Multivariate		
Parameter	Odds Ratio	95% CI	P	Odds Ratio	95% CI	P
Age (≥54 years)	4.00	1.77,9.03	0.001	5.23	1.65,16.54	0.005
Sex (Female)	0.65	0.29,1.46	0.294			
BMI (≥23)	1.06	0.48,2.31	0.891			
Ascites	1.10	1.01,1.19	0.035			
The distance between transducer and Glisson capsule (mm) (≥16)	2.10	0.96,4.59	0.062			
The distance between Glisson capsule and ROI(mm) (≥16)	0.90	0.42,1.94	0.788			
Fibrosis staging (≥F2)	38.44	10.44,141.56	<0.001	21.69	2.90,162.47	0.003
Steatosis (≥S3)	1.54	0.42,5.61	0.511			
Necroinflammation (≥A3)	1.04	0.99,1.09	0.190			
Chronic hepatitis B	20.63	5.73,74.29	<0.001			

LS, liver stiffness; CI, confidence interval; BMI, body mass index; ROI, region-of-interest

### Diagnostic performance of 2D CP-SWE in differentiating fibrosis staging

**Fig 5:**
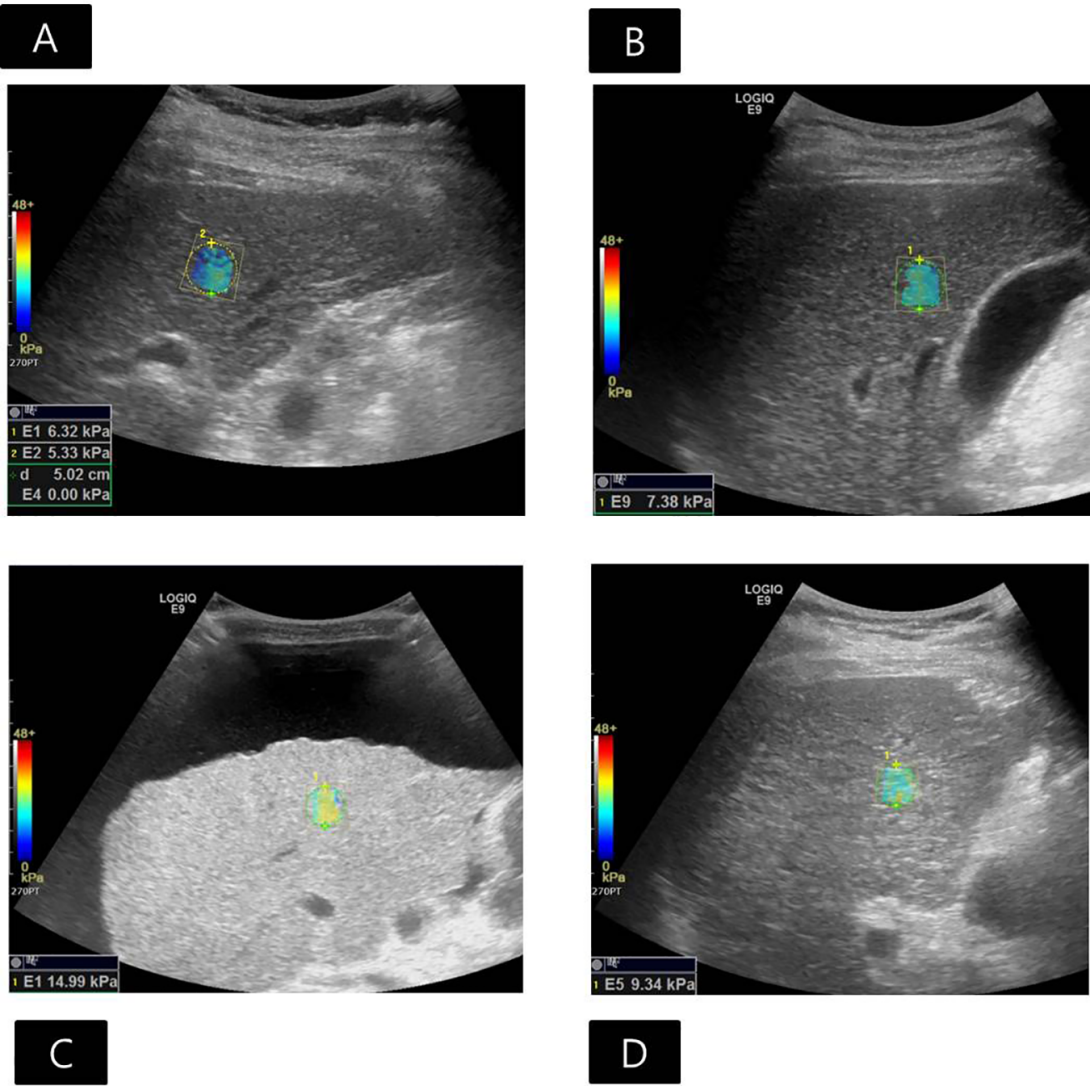
When applying the cutoff values from our study, the live stiffness (LS) values obtained by the 2D comb-push shear wave elastography (2D CP-SWE) could predict histologic fibrosis staging. **A)**The median LS value of 5.98kPa was achieved by the 2D CP-SWE in a 64-year-old man, which was greater than the cutoff value of F2 (5.33kPa) and less than the cutoff value of F3 (6.84kPa). The patient underwent surgical resection for hepatocellular carcinoma (HCC), after which the fibrosis staging was histologically confirmed to be F2. **B)** The median LS value of 7.38kPa was measured by the 2D CP-SWE in a 66-year-old man, which was greater than the cutoff value of F3 (6.84kPa) and less than the cutoff value of F4 (7.59kPa). The patient underwent surgical resection for HCC, after which the fibrosis stage was histologically confirmed as F3. **C)** Despite massive ascites, reliable LS values were successfully measured by 2D CP-SWE in a 57-year-old woman. The median LS value of 13.45kPa was greater than the cutoff value of F4 (7.59kPa). The patient underwent liver transplantation. The fibrosis stage was histologically proven to be F4, and the macronodular cirrhosis was associated with alcohol consumption. **D)** Reliable LS values were successfully measured by 2D CP-SWE in a 49-year-old obese man with a body mass index of 31.33kg/m^2^. The median LS value of 8.14kPa was greater than the value of F4 (7.59kPa). The patient underwent liver transplantation. The fibrosis stage was histologically proven to be F4, and macronodular cirrhosis was associated with hepatitis B virus infection and alcohol consumption.

According to ROC analysis, the AUC of LS values at 2D CP-SWE for fibrosis stage F2 or greater was 0.874 (95% CI: 0.794–0.930), with an optimal cutoff of 5.33kPa yielding 93.2% sensitivity and 73.8% specificity (Table 4). The ROC curve revealed that AUCs of LS values for stage F3 or greater and stage F4 fibrosis were 0.905 (95% CI: 0.832–0.954, Fig 5) and 0.894 (95% CI: 0.819–0.946), respectively. The adjusted AUCs in the diagnosis of stage F2 or greater, stage F3 or greater, and F4 were 0.836, 0.867, and 0.856, respectively. The overall accuracy of 2D CP-SWE for differentiating fibrosis staging was represented as 0.931 as the Obuchowski measurement.

**Table 4:** Diagnostic performance of 2D CP-SWE for fibrosis staging (n = 105).

Parameter	≥F1	≥F2	≥F3	F4	Obuchowski Measurement
AUC	0.853	0.874	0.905	0.894	0.931
adjusted AUC	0.815	0.836	0.867	0.856	
95% CI	0.771,0.915	0.794,0.930	0.832,0.954	0.819,0.946	
optimal cutoff value (kPa)	5.18	5.33	6.84	7.59	
Sensitivity(%)	80.3	93.2	84.4	83.3	
Specificity(%)	79.6	73.8	82.2	85.1	

2D CP-SWE, 2-dimensional comb-push shear wave elastography; AUC, area under the curve

## Discussion

Our results suggested that the 2D CP-SWE was a reliable noninvasive method for assessing hepatic fibrosis, with an overall accuracy of 0.931 as the Obuchowski measurement. The AUCs of LS measurements using 2D CP-SWE for fibrosis stage F≥2, F≥3, and F4 in our study were 0.874, 0.905, and 0.894, respectively.

In our study, LS values highly correlated with histologic fibrosis staging and slightly correlated with necroinflammation using the Spearman correlation. Multiple regression analysis showed that fibrosis staging (β = 1.123, P<0.001) was an independent factor affecting LS values. Our results were in good agreement with the results of a previous study with 2D-SWE, which found that LS values were not influenced by steatosis and necroinflammation [31]. In addition, according to our univariate analysis regarding the factors affecting LS value, age > 54 years (OR 4.0, 95% CI 1.78–9.03, P = 0.001), presence of ascites (OR 1.1, 95% CI 1.01–1.19, P = 0.035), fibrosis staging greater than F2 (OR 38.4, 95% CI 10.44–141.56, P<0.001), and HBV-CLD (OR 20.6, 95% CI 5.73–74.29) were significantly associated with the high LS values (>5.33kPa). However, steatosis, BMI, the distance between transducer and liver capsule were not significant factors associated with high LS values. Furthermore, in our multivariate analysis, only fibrosis staging greater than F2 (OR 21.7, 95% CI 2.90–162.47, P = 0.003) remained as an independent predictor for the high LS values (>5.33kPa). Therefore, based on our study results, the LS values measured with the new 2D-SWE using comb-push technique were directly related to the liver fibrosis stage, not being confounded by other potential factors.

Our results of diagnostic performance with the 2D CP-SWE were similar to the results of the previous meta-analysis studies using TE and pSWE. The AUCs for hepatic fibrosis stage F≥2, F≥3, and F4 were 0.874, 0.905, and 0.894, respectively, in a meta-analysis study using TE, reported by Friedrich et al.[36]. A meta-analysis of the value of pSWE demonstrated that the AUCs for hepatic fibrosis stage F≥2, F≥3, and F4 were 0.84, 0.89, and 0.91, respectively[37]. In addition, the diagnostic performance of 2D CP-SWE in the diagnosis of fibrosis stage F≥2 was similar to that of SSI 2D-SWE in several previous studies [17, 28, 31, 38]. However, when the Obuchoswki measure was applied, the overall diagnostic performance of the 2D CP-SWE (0.931) was higher than that of SSI 2D-SWE (0.807) [32]. To the best of our knowledge, our study is the first study reporting the diagnostic performance of the innovated 2D CP-SWE using histology as the reference standard. Based on our findings, we believe that the new 2D CP-SWE can provide diagnostic accuracy comparable to that of the currently available US elastography techniques for fibrosis staging.

In our study, the applicability rate, technical failure rate, and unreliability rate of the 2D CP-SWE were 90.8%, 0.8%, and 8.35%, respectively. Although the applicability of liver elastography can be affected by the characteristics of the patients enrolled in the study, the applicability rate of 2D CP-SWE in our study was within the range of that of SSI 2D-SWE (89.6%–94.9%) [38, 39], and pSWE (78.49%–98.7%) [38–40], and it was higher than that of TE (81.1%–85.7%) [21, 41]. The technical failure rate in our study was lower than that of SSI 2D-SWE (2.3–10.4%) in some studies [17, 28, 38], which suggested 2D CP-SWE had advantage of technical success. Our study suggested that the new 2D CP-SWE was a stable method, with a relatively high technical success rate.

Previous studies reported that the technical failure of TE and other SWE was influenced by age, ascites, BMI, and diabetes [1, 21, 38, 40]. Similarly, patients with non-applicable 2D CP-SWE in our study had an older age, higher incidence of ascites, and greater distance between the transducer and Glisson capsule. However, BMI was not significantly different between patients with applicable 2D CP-SWE and patients with non-applicable 2D CP-SWE. One technical failure in our study was observed in patients with normal BMI and long distance between the transducer and Glisson capsule. The distance between the transducer and Glisson capsule might account for the unsuccessful SWE in patients with a high BMI. The long distance could suggest greater adipose tissue between the skin and the liver capsule, which increases the absorption of acoustic energy and decreases the magnitude of the applied radiation force [38]. Of interest to note is that in our study technical failure was not observed in patients with ascites or obesity. It could be explained by the fact that the distance between transducer and Glisson capsule is also lengthened by omental fat or intervening colon. Therefore, we should pay more attention to LS measurement if thick omental fat or intervening colon is present. The effort to reduce the distance between transducer and Glisson capsule might increase the applicability rate of SWE.

Our result revealed excellent intraobserver repeatability with an ICC of 0.95 and interobserver reproducibility with an ICC of 0.87 of a new 2D CP-SWE, which was comparable with those of TE and SSI 2D-SWE [28, 42]. Considering the invasiveness and potential complications of percutaneous liver biopsy for fibrosis staging, one of the promising applications of SWE would be noninvasive monitoring of the development of liver fibrosis or cirrhosis in patients with chronic liver diseases, or monitoring of the therapeutic effects of antiviral or antifibrotic agents [1, 5, 11, 12]. In this regard, to replace histologic assessment of fibrosis staging by SWE, SWE techniques should have excellent intra- and interobserver reproducibility. Our results of intraobserver reproducibility of the 2D CP-SWE technique were similar to a previous study regarding 2D CP-SWE, which reported an ICC of 0.995, but the results of inter-observer reproducibility were slightly inferior to the previous study reporting an ICC of 0.991[30]. The difference in ICCs might be attributed to the different study populations. Only five patients who were presumed to have fibrosis of stage F4 based on MRE among a total of 47 patients were included in the research by Song et al. [30], while 18 patients with cirrhosis confirmed by histopathology in a total of 120 patients were included in our study.

According to the European Association for the Study of the Liver (EASL) guidelines, all patients with chronic HCV infection should be screened with TE as the evaluation of hepatic fibrosis is important to determine the treatment policy and predict the prognosis of patients with chronic liver diseases [5]. However, several studies have demonstrated that the application of TE in patients with obesity or ascites has limitations [19, 21]. Moreover, the 2D CP-SWE provides an advantage of larger sample volume than that afforded by TE technique [18]. Furthermore, 2D CP-SWE has advantages over TE in that the 2D CP-SWE uses the same probe as that used in the B-mode image, enabling avoidance of artifact and vessels, and screening for the development of hepatocellular nodules or HCC [1, 26]. Considering the high applicability and comparable diagnostic performance of 2D CP-SWE to TE for staging of hepatic fibrosis, we expect that 2D CP-SWE in combination of B-mode ultrasound evaluation on a single US system may provide an advantage of a “one-stop shopping” modality compared with combined use of TE and B-mode ultrasound for patients with chronic hepatitis B or C infection. In addition, the 2D CP-SWE displays the colored elasticity maps, allowing instant judgment on the validity and facilitates larger ROIs than pSWE techniques, similar to SSI 2D-SWE. Moreover, the 2D CP-SWE has an advantage of being implementable on conventional US system and has a faster frame rate of elastograms than SSI 2D-SWE, which may allow faster LS measurements in a cine-loop time acquisition [43]. Its faster frame rate is explained by the fact that comb-push ultrasound shear elastography methods produce and process multiple shear waves to reconstruct a full field-of-view (FOV) with only single acquisition, while SSI generates one shear wave from multiple focused pushes per one acquisition and requires multiple push-detection acquisitions to reconstruct a full FOV map [29, 43]. Moreover, time-aligned sequential tracking allows high pulse-repetition-frequency with parallel beamforming capability on conventional diagnostic ultrasound system by sequentially firing tracking vectors and aligning shear wave data in the temporal direction [44]. In fact, SSI 2D-SWE is currently a quite well-established SWE technique for the staging of fibrosis but requires software beamformers enabling high pulse-repetition-frequency for tracking capability through the use of synthetic aperture imaging. However, as the majority of current clinical ultrasound systems do not have this software beamforming capability, it is a critical hurdle for translating 2D SWE into mainstream ultrasound system [26].

Our study had several limitations. First, our study population was small, and the underlying liver disease was disproportionate. Our study population was too small to evaluate the applicability of comb-push 2D-SWE and the factors affecting its applicability. Most patients with chronic viral liver diseases in our study were infected with HBV, while only three patients infected with HCV were included. Further studies are warranted, since the etiology may affect LS cutoff value [5]. Second, we did not take the pre-probability of cirrhosis into account when we selected the cutoff value, although we adjusted AUC for the diagnostic performance for hepatic fibrosis staging to reduce spectral bias. Finally, interobserver reproducibility was evaluated in 20 volunteers without a histologic diagnosis.

In conclusion, 2D CP-SWE can be a useful and reliable method to access liver fibrosis in a noninvasive manner. Its applicability might be influenced by age, ascites, and the distance between the transducer and Glisson capsule.

## Supporting information

S1 Table Comparison in patients with ascites according to the applicability of 2D CP-SWE. (DOCX)

S2 Table Reasons for histologic sampling and histologic diagnosis in patients enrolled in this study. (DOCX)

S3 Table Fibrosis staging and etiology of chronic hepatitis in patients with chronic hepatitis (n = 54). (DOCX)

S4 Table Correlation of liver stiffness value and BMI in each fibrosis stage. (DOCX)
