# Supplementary material for: Liver fibrosis staging with a new 2D-shear wave elastography using comb-push technique: Applicability, reproducibility, and diagnostic performance
Source: PLoS One. 2017 May 16;12(5):e0177264. doi: 10.1371/journal.pone.0177264 (PMC5433696; doi:10.1371/journal.pone.0177264)
Supplement: S1 Table — (DOCX) [file pone.0177264.s001.docx]

**S1 Table. Comparison in patients with ascites according to the applicability of 2D CP-SWE**

|  |  | Applicable 2D CP-SWE in patient with ascites  (n=6) | Non-applicable 2D CP-SWE in patients with ascites  (n=6) | P value |
| --- | --- | --- | --- | --- |
| Mean age |  | 57.2 age | 58.5 agei | 0.423 |
| Sex* | M | 5 (83.3%) | 3 (50.0%) | 0.545 |
|  | F | 1 (16.7%) | 3 (50.0%) |  |
| Mean BMI(kg/㎡) |  | 21.3 BMI | 24.7 BMI | 0.262 |
| The distance between transducer  and Glisson capsule (mm) |  | 26.1sule | 36.2sule | 0.262 |
| The distance between Glisson capsule  and ROI (mm) |  | 18.4d RO | 14.5d RO | 0.2 |

2D CP-SWE, 2-dimensional comb-push shear wave elastography; BMI, body mass index; ROI, region-of-interest

Note.−Unless otherwise indicated, data are means ± standard deviations.

*Data are numbers of patients, and data in parentheses are percentages.
