# Supplementary material for: Liver fibrosis staging with a new 2D-shear wave elastography using comb-push technique: Applicability, reproducibility, and diagnostic performance
Source: PLoS One. 2017 May 16;12(5):e0177264. doi: 10.1371/journal.pone.0177264 (PMC5433696; doi:10.1371/journal.pone.0177264)
Supplement: S2 Table — (DOCX) [file pone.0177264.s002.docx]

**S2 Table. Reasons for histologic sampling and histologic diagnosis in patients enrolled in this study**

| Reasons for histologic sampling | Histologic diagnosis |
| --- | --- |
| **Liver biopsy (n=44)** |  |
| Follow-up of transplantation (n=20) | No evidence of acute rejection (n=15) |
|  | Acute cellular rejection (n=4) |
|  | Mild cholangitis (n=1) |
| Elevated liver function test (n=22) | Primary biliary cirrhosis (n=6) |
|  | Autoimmune hepatitis (n=4) |
|  | Steatohepatitis, NASH (n=4) |
|  | Acute hepatitis (n=2) |
|  | Chronic hepatitis (n=1) |
|  | Toxic hepatitis (n=1) |
|  | Viral hepatitis (n=1) |
|  | Secondary hepatitis due to systemic infection(n=1) |
|  | Nonspecific (n=2) |
| Screening for Wilson's disease due to family history (n=2) | No evidence of Wilson's disease (n=2) |
| **Hepatectomy for liver donation (n=23)** | Normal parenchyme in a liver donor (n=21) |
|  | Simple steatosis (n=2) |
| **Explantation for liver transplantation (n=20)** | Macronodular cirrhosis with HCCs (n=9) |
|  | Macronodular cirrhosis (n=5) |
|  | Macronodular cirrhosis with totally necrotic nodules (n=3) |
|  | Chronic hepatitis with HCCs (n=1) |
|  | Chronic hepatitis with totally necrotic nodules (n=1) |
|  | Wilson's disease (n=1) |
| **Hepatic resection for hepatic tumor (n=27)** | HCC (n=19) |
|  | Combined HCC-CCC (n=4) |
|  | CCC (n=1) |
|  | Intraductal papillary neoplasm with high grade dysplasia (n=1) |
|  | HCC and CCC (n=1) |
|  | HCC and combined HCC-CCC (n=1) |

NASH, nonalcoholic steatohepatitis; HCC, hepatocellular carcinoma; combined HCC-CCC, combined hepatocellular and cholangiocarcinoma; CCC, cholangiocarcinoma
