# Supplementary material for: Liver fibrosis staging with a new 2D-shear wave elastography using comb-push technique: Applicability, reproducibility, and diagnostic performance
Source: PLoS One. 2017 May 16;12(5):e0177264. doi: 10.1371/journal.pone.0177264 (PMC5433696; doi:10.1371/journal.pone.0177264)
Supplement: S3 Table — (DOCX) [file pone.0177264.s003.docx]

**S3 Table. Fibrosis staging and etiology of chronic hepatitis in patients with chronic hepatitis (n=54)**

| Fibrosis staging | Cause of chronic hepatitis |
| --- | --- |
| F0 (n=6) | NASH (n=2) |
|  | HBV and NASH (n=1) |
|  | PBC (n=2) |
|  | Autoimmune (n=1) |
| F1 (n=5) | HBV (n=1) |
|  | NASH (n=1) |
|  | PBC (n=3) |
| F2 (n=11) | HBV (n=9) |
|  | HCV (n=1) |
|  | HCV and alcohol (n=1) |
| F3 (n=14) | HBV (n=11) |
|  | HCV (n=1) |
|  | Autoimmune (n=1) |
|  | Idiopathic (n=1) |
| F4 (n=18) | HBV (n=13) |
|  | Alcohol (n=2) |
|  | HBV and alcohol (n=1) |
|  | Idiopathic (n=2) |

NASH, nonalcoholic steatohepatitis; HBV, hepatitis B virus; PBC, primary biliary cirrhosis; HCV, hepatitis C virus
