# Supplementary material for: Liver fibrosis staging with a new 2D-shear wave elastography using comb-push technique: Applicability, reproducibility, and diagnostic performance
Source: PLoS One. 2017 May 16;12(5):e0177264. doi: 10.1371/journal.pone.0177264 (PMC5433696; doi:10.1371/journal.pone.0177264)
Supplement: S4 Table — (DOCX) [file pone.0177264.s004.docx]

**S4 Table. Correlation of liver stiffness value and BMI in each fibrosis stage**

|  | F0 | F1 | F2 | F3 | F4 |
| --- | --- | --- | --- | --- | --- |
| Correlation coeffient,r | -0.035 | -0.357 | -0.278 | -0.0957 | -0.484 |
| Significance level | P=0.821 | P=0.160 | P=0.382 | P=0.745 | P=0.042 |
| 95% CI for r | (-0.329,0.265) | (-0.715,0.150) | (-0.735,0.352) | (-0.596,0.458) | (-0.776,0.022) |

BMI, body mass index; CI, confidence interval
